# N-terminal pro-B-type natriuretic peptide as a prognostic indicator for 30-day mortality following out-of-hospital cardiac arrest: a prospective observational study

**DOI:** 10.1186/s12872-020-01630-x

**Published:** 2020-08-24

**Authors:** Reidun Aarsetøy, Torbjørn Omland, Helge Røsjø, Heidi Strand, Thomas Lindner, Hildegunn Aarsetøy, Harry Staines, Dennis W. T. Nilsen

**Affiliations:** 1 Department of Clinical Science, Faculty of Medicine, University of Bergen, Bergen, Norway; 2 Department of Cardiology, Division of Medicine, Stavanger University Hospital, Mailbox 8100, 4068 Stavanger, Norway; 3 Institute for Clinical Medicine, Faculty of Medicine, University of Oslo, Oslo, Norway; 4 Department of Cardiology, Division of Medicine, Akershus University Hospital , Lørenskog, Norway; 5 Division of Research and Innovation, Akershus University Hospital, Lørenskog, Norway; 6 Multidisciplinary Laboratory Medicine and Medical Biochemistry, Akershus University Hospital, Lørenskog, Norway; 7 The Regional Centre for Emergency Medical Research and Development (RAKOS), Stavanger University Hospital , Stavanger, Norway; 8 Department of Endocrinology, Division of Medicine, Stavanger University Hospital, Stavanger, Norway; 9 Sigma Statistical Services, Sigma Statistical Services, Balmullo, UK

**Keywords:** Out-of-hospital cardiac arrest, Prognosis, High-sensitivity cardiac troponin T, Copeptin, N-terminal pro-B-type natriuretic peptide

## Abstract

**Background:**

Early risk stratification applying cardiac biomarkers may prove useful in sudden cardiac arrest patients. We investigated the prognostic utility of early-on levels of high sensitivity cardiac troponin-T (hs-cTnT), copeptin and N-terminal pro-B-type natriuretic peptide (NT-proBNP) in patients with out-of-hospital cardiac arrest (OHCA).

**Methods:**

We conducted a prospective observational unicenter study, including patients with OHCA of assumed cardiac origin from the southwestern part of Norway from 2007 until 2010. Blood samples for later measurements were drawn during cardiopulmonary resuscitation or at hospital admission.

**Results:**

A total of 114 patients were included, 37 patients with asystole and 77 patients with VF as first recorded heart rhythm. Forty-four patients (38.6%) survived 30-day follow-up. Neither hs-cTnT (*p* = 0.49), nor copeptin (*p* = 0.39) differed between non-survivors and survivors, whereas NT-proBNP was higher in non-survivors (*p* <  0.001) and significantly associated with 30-days all-cause mortality in univariate analysis, with a hazard ratio (HR) for patients in the highest compared to the lowest quartile of 4.6 (95% confidence interval (CI), 2.1–10.1), *p* <  0.001. This association was no longer significant in multivariable analysis applying continuous values, [HR 0.96, (95% CI, 0.64–1.43), *p* = 0.84]. Similar results were obtained by dividing the population by survival at hospital admission, excluding non-return of spontaneous circulation (ROSC) patients on scene [HR 0.93 (95% CI, 0.50–1.73), *P* = 0.83]. We also noted that NT-proBNP was significantly higher in asystole- as compared to VF-patients, *p* <  0.001.

**Conclusions:**

Early-on levels of hs-cTnT, copeptin and NT-proBNP did not provide independent prognostic information following OHCA. Prediction was unaffected by excluding on-scene non-ROSC patients in the multivariable analysis.

**Trial registration:**

ClinicalTrials. gov, NCT02886273.

## Background

Out-of-hospital cardiac arrest (OHCA) is a leading cause of death and represents a major health problem in western countries. In Europe, the annual incidence of Emergency Medical Services (EMS) attended OHCA is estimated to 84 per 100,000 inhabitants [1]. The mortality rate is high, with an overall rate of survival to hospital discharge of only 10% [1, 2]. Early and precise risk stratification of cardiac arrest patients is important to guide further treatment decisions. On-scene observations [3] and 24-h intensive care follow-up recordings [4, 5] are currently being used to predict survival from OHCA.

Heart disease is a major risk factor for sudden cardiac arrest [6], with coronary artery disease and cardiomyopathies as the two most common causes of sudden cardiac death [6, 7]. Cardiac arrest with global ischemia and subsequent resuscitation may contribute to myocardial injury and dysfunction [8, 9]. Cardiac troponin (cTn) and copeptin are biomarkers used for early diagnosis of acute myocardial infarction (AMI) [10, 11] and may also serve as prognostic indicators following an acute coronary syndrome (ACS) [10, 12]. N-terminal pro-B-type natriuretic peptide (NT-proBNP) is a well-established diagnostic marker of heart failure [13] and an important predictor of mortality in patients with heart failure [13] or ACS [14]. Both NT-proBNP [15, 16] and copeptin [17, 18] have been found to predict mortality in critically ill patients admitted to a medical intensive care unit (ICU).

Although cardiac biomarkers may be useful for prognostication in OHCA-patients, available data regarding the predictive value of high-sensitivity cardiac troponin-T (hs-cTnT) [9, 19–21], copeptin [21–23] and NT-proBNP [19, 21, 24, 25] diverge. The inconsistency in previously reported results may be related to heterogeneity in patient population regarding cause of cardiac arrest and primary heart rhythm, timing of blood sampling and follow-up time. Previous studies have also mainly focused on resuscitated patients admitted to the ICU.

In our study, we included patients with OHCA of assumed cardiac origin. In a previous publication [21], we have assessed the prognostic utility of hs-cTnT, copeptin and NT-proBNP in OHCA patients with ventricular fibrillation (VF). We also compared ischemic- with non-ischemic cardiac arrest. The primary aim of the present study was to evaluate whether these three biomarkers may serve as independent prognostic indicators in OHCA patients with shockable- and non-shockable heart rhythm, adjusting for clinical variables related to cardiac arrest. As a second aim, this study was performed to improve our understanding of pathophysiology related to OHCA.

## Methods

### Study subjects and design

From February 2007 until November 2010 we collected blood samples from patients ≥18 years of age with OHCA of assumed cardiac origin, defined according to the Utstein definitions [26], in the southwestern part of Norway. All patients recruited in this study received out-of-hospital advanced cardiac life support according to the 2005 European Resuscitation Council guidelines with Norwegian modifications [27]. Blood sampling was performed by the EMS paramedics, 20 ml of blood was collected into ethylenediamine tetra acetic acid (EDTA) -tubes, using a venous cannula, during or immediately after termination of cardiopulmonary resuscitation (CPR). In patients with return of spontaneous circulation (ROSC) without a prehospital blood sample, blood was collected immediately after hospital admission.

We applied clinical information from hospital records and collected additional information from electrocardiograms, echocardiography and coronary angiography, to confirm an assumed cardiac cause of OHCA and to categorize patients [21]. OHCA-patients were divided into two groups according to first recorded heart rhythm, asystole or VF. VF-patients were further categorized according to an acute ischemic or non-ischemic mechanism for sudden cardiac arrest, and whether or not they had previously known heart disease.

Informed consent was collected retrospectively. All survivors gave written, informed consent. If the patient did not regain consciousness before death, the next-of-kin were asked for consent on the patient’s behalf. This study was approved by the Regional Board of Research Ethics and the Norwegian Health Authorities, conducted in accordance with the Helsinki Declaration of 1975, as revised in 1989, and registered in ClinicalTrials.gov, identifier: NCT02886273.

### Laboratory methods

After collection, blood samples were centrifuged at 2500 rpm for 10 min. Within 24 h if stored at room-temperature, or within 48 h if stored at + 4 °C. EDTA-plasma was stored in aliquots at − 70 °C. Measurements of hs-cTnT, copeptin and NT-proBNP were performed at the Department of Multidisciplinary Laboratory Medicine and Medical Biochemistry, Akershus University Hospital, applying standardized methods, as previous reported [21]. Hs-cTnT (according to local stability analyses) and NT-proBNP [28] are found to be stable in EDTA-plasma for up to 3 days stored at room-temperature, and even longer when stored at + 4 °C. Ex vivo copeptin stability in EDTA-plasma is shown for at least 7 days at room temperature and 14 days at + 4 °C [29].

### Statistical methods

Descriptive statistics are presented as medians with interquartile range (IQR; 25th to 75th percentile) for continuous data and as numbers and percentages for categorical data. Differences in baseline characteristics were assessed by the Kruskal-Wallis Test for continuous data and Fisher’s exact test for categorical data. Due to a right-skewed distribution, hs-cTnT, copeptin and NT-proBNP levels were logarithmically transformed to the base-e (log_e_) prior to analysis. Student’s independent two-sample t-test was used after log_e_-transformation to assess between-group differences, comparing biomarker-levels in non-survivors with survivors, between the asystole- and VF-group, and between patients who died on scene and those surviving until hospital admission. Spearman’s rank correlation coefficient was calculated to identify a possible relation between biomarker levels and time of blood sampling.

Patients were divided into quartiles (Q1–4) according to the hs-cTnT, copeptin and NT-proBNP concentrations. The Kaplan-Meier product limits were used for plotting the times to event and the log-rank test was used to test for the equality of the survival curves. Cox regression models, applying both quartiles and continuous log_e_-transformed values, were fitted for each of the biomarkers for the analysis of all-cause mortality within 30-days. We employed two different models for the multivariable analysis, by including age, log_e_-creatinine, VF as first recorded heart rhythm, and duration of resuscitation for Model 1, and adding gender, witnessed cardiac arrest and bystander-initiated CPR for Model 2. Hazard ratios (HR) with 95% confidence intervals were calculated for each of the higher quartiles as compared to quartile 1. For continuous log_e_-transformed values, we employed HR and 95% CI per standard deviation (SD) increase of the biomarkers. Subgroup analyses were performed in survivors at hospital admission and for VF-patients.

Statistical analysis was performed using the statistical package SPSS version 25 (IBM Corp. Armonk, NY). All tests were 2 -sided with a significance level of 5% without multiplicity adjustment.

## Results

**Fig. 1:**
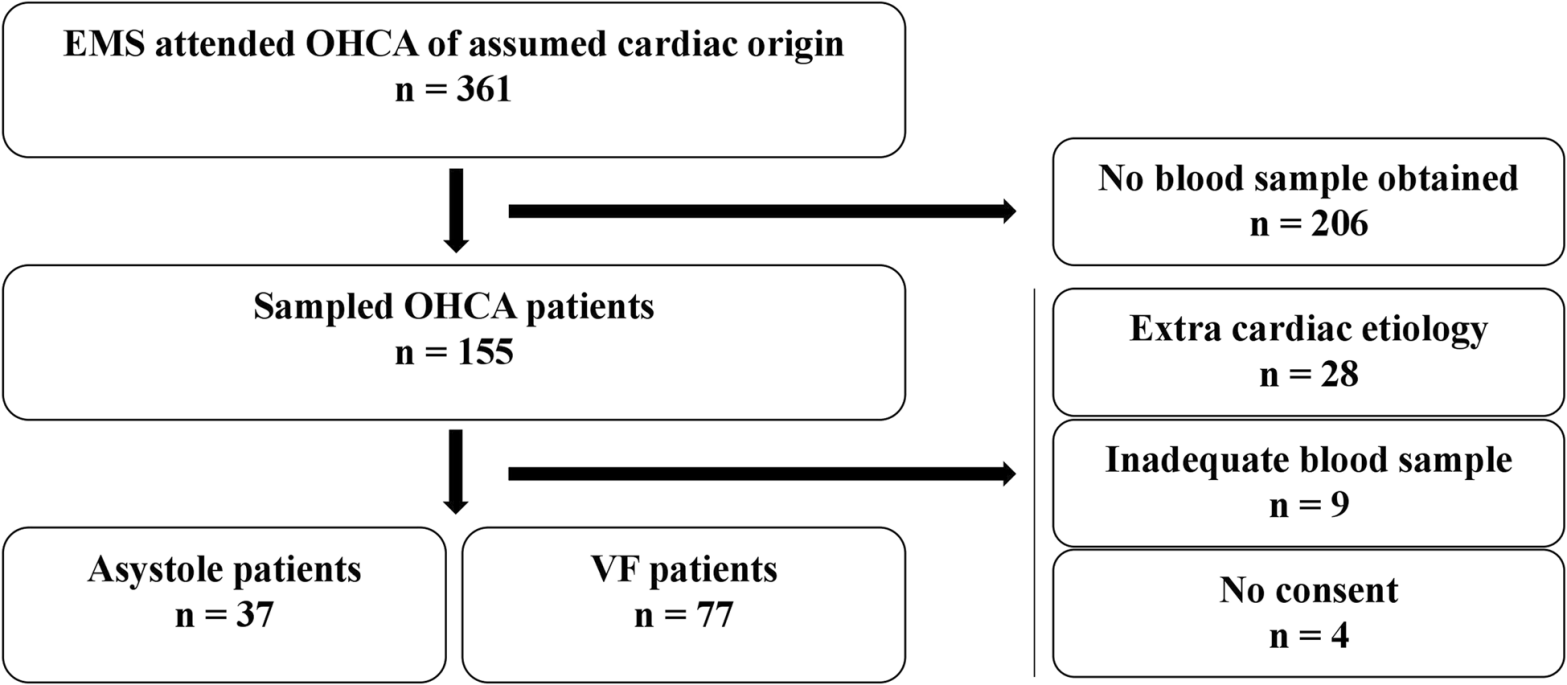
Flow chart displaying selection and classification of patients with out-of-hospital cardiac arrest recruited between February 2007 and November 2010

During the study period, 361 patients suffered cardiac arrest of assumed cardiac origin, and were eligible for inclusion in this study [21]. Out of these, 155 patients were sampled. Retrospectively, a total of 41 patients had to be excluded for different reasons (Fig. 1). The final population of 114 OHCA-patients was further divided into two groups according to the first recorded heart rhythm; 37 patients with asystole and 77 patients with VF. The group of VF-patients was further classified according to the presence of an acute ischemic event, by which 53 patients had signs of an AMI [21], and 10 of these had previously been diagnosed with heart disease. Twenty-one patients suffered sudden cardiac arrest without signs of an AMI [21], of whom 18 had evidence of prior heart disease, including coronary artery disease and/or chronic heart failure. Due to lack of clinical information, three patients could not be classified as having an ischemic- or non-ischemic cardiac arrest.

The baseline characteristics of the patients stratified according to 30-day mortality are presented in Table 1. Out of 114 patients, 70 (61.4%) died on scene or before hospital discharge. All patients (38.6%) surviving to hospital discharge were still alive at 30-day follow-up. Compared to 30-day survivors, non-survivors were older, had increased prevalence of heart failure and diabetes and worse kidney function. Asystole as first recorded heart rhythm was more frequent in non-survivors (53%), they also had worse cardiac arrest conditions with a significant lower proportion of witnessed cardiac arrest and bystander-initiated CPR, and longer duration of resuscitation.

**Table 1:** Baseline characteristics and laboratory values of patients suffering out-of-hospital cardiac arrest

	**Total population (*n*** **= 114)**	**Survivors (*n*** **= 44)**	**Non-survivors (*n*** **= 70)**	***P*-value**
Age, y	67 (56–78)	61 (51–68)	72 (62–83)	< 0.001
Male gender	95 (83)	36 (82)	59 (84)	0.80
**Previous history**				
Angina pectoris	17 (18)	6 (14)	11 (22)	0.42
Myocardial infarction	33 (31)	10 (23)	23 (36)	0.20
Previous PCI	12 (11)	5 (11)	7 (11)	1.00
Previous CABG	12 (11)	2 (5)	10 (16)	0.12
Heart failure	28 (26)	7 (16)	21 (34)	0.046
Hypertension	53 (52)	19 (43)	34 (58)	0.17
Diabetes mellitus	17 (15)	2 (5)	15 (25)	0.007
Hypercholesterolemia	44 (42)	22 (51)	22 (36)	0.16
Smoking				0.81
Current smoker	27 (32)	13 (33)	14 (31)	
Ex-smoker	40 (47)	20 (50)	20 (44)	
**Cardiac arrest conditions**				
Witnessed cardiac arrest	87 (77)	39 (89)	48 (70)	0.022
Bystander-initiated CPR	93 (82)	40 (91)	53 (76)	0.049
Duration of resuscitation, min	23 (10–38)	9 (5–15)	32.5 (23–45)	< 0.001
Initial rhythm				< 0.001
VF	77 (68)	44 (100)	33 (47)	
Asystole	37 (33)	0 (0)	37 (53)	
**Baseline blood samples**				
Creatinine (μmol/L)	102 (87–122)	93 (82–116)	110 (92–125)	0.013
Total cholesterol (mmol/L)	4.2 (3.6–5.3)	4.9 (4.1–6.5)	3.9 (3.4–5.0)	0.002
CRP (mg/L)	2.5 (1.1–9.9)	2.1 (1.2–4.6)	3.9 (1.0–18.0)	0.15
Glucose (mmol/L)	12.6 (8.0–16.9)	12.6 (8.8–14.7)	12.6 (6.6–17.8)	0.94
Copeptin (pmol/L)^a^	436 (216–825)	388 (195–825)	445 (244–879)	0.39
hs-cTnT (ng/L)^b^	71 (26–231)	100 (21–289)	66 (26–207)	0.49
NT-proBNP (pmol/L)^c^	61 (25–234)	30 (12–98)	105 (35–495)	< 0.001

Data are presented as median (interquartile range) or numbers (%)

^a^ *n* = 110 (96%), ^b^ *n* = 111 (97%), ^c^ *n* = 112 (98%)

*Abbreviations*: *CPR* cardiopulmonary resuscitation, *PCI* percutaneous coronary intervention, *CABG* coronary artery bypass graft, *CRP* C-reactive protein, *hs-cTnT* high-sensitivity cardiac troponin T, *NT-proBNP* N-terminal pro-B-type natriuretic peptide

Blood samples were drawn from 75 patients during resuscitation and from 39 patients at hospital admission, with a median time from cardiac arrest until blood sampling of 31 and 73.5 min, respectively. We found no correlation between biomarker levels and time of blood sampling, except for hs-cTnT (*r* = 0.31, *p* = 0.001). Median hs-cTnT concentration was 71 (IQR; 26–231) ng/L, ranging from 6.0 to 8333 ng/L. Hs-cTnT levels did not differ significantly between survivors and non-survivors [median 100 IQR: (21–289) ng/L vs. 66 (26–207) ng/L, *p* = 0.49]. Kaplan-Meier analysis revealed no significant association between quartiles of hs-cTnT and all-cause mortality at 30-days follow-up (log-rank *p* = 0.57). In multivariable analysis, there were no significant associations between hs-cTnT and 30-days all-cause mortality, neither in the total population, nor for survivors at hospital admission or in VF-patients (Table 2).

**Table 2:** Univariate- and multivariable Cox regression models applying continuous log_e_-transformed values of hs-cTnT, copeptin and NT-proBNP

	**Univariate**		**Multivariable**			
			**Model 1**		**Model 2**	
	**HR (95% CI)**	***P*-value**	**HR (95% CI)**	***P*-value**	**HR (95% CI)**	***P*-value**
**Total population**						
log_e_ Hs-cTnT	0.99 (0.78–1.29)	0.99	0.87 (0.66–1.15)	0.34	0.90 (0.68–1.19)	0.46
log_e_ Copeptin	1.08 (0.84–1.39)	0.54	0.88 (0.65–1.20)	0.42	0.92 (0.66–1.28)	0.62
log_e_ NT-proBNP	1.86 (1.44–2.41)	< 0.001	0.96 (0.64–1.43)	0.84	0.88 (0.57–1.36)	0.57
**Patients surviving to hospital admission**						
log_e_ Hs-cTnT	0.96 (0.67–1.38)	0.83	0.85 (0.58–1.25)	0.41	0.87 (0.61–1.26)	0.48
log_e_ Copeptin	1.40 (0.95–2.06)	0.093	1.20 (0.75–1.93)	0.45	1.07 (0.62–1.83)	0.81
log_e_ NT-proBNP	1.86 (1.24–2.78)	0.003	0.93 (0.50–1.73)	0.83	0.79 (0.41–1.49)	0.46
**VF-patients**						
log_e_ Hs-cTnT	0.85 (0.59–1.24)	0.41	0.78 (0.52–1.17)	0.24	0.85 (0.56–1.28)	0.43
log_e_ Copeptin	1.15 (0.81–1.63)	0.44	1.01 (0.67–1.51)	0.98	0.95 (0.60–1.52)	0.85
log_e_ NT-proBNP	1.67 (1.07–2.60)	0.023	0.84 (0.43–1.64)	0.61	0.51 (0.22–1.19)	0.12

*Abbreviations*: *log*_*e*_ *hs-cTnT* log_e_-transformed value of hs-cTnT, *log*_*e*_ *Copeptin* log_e_-transformed value of copeptin, *log*_*e*_ *NT-proBNP* log_e_-transformed value of N-terminal pro B-type natriuretic peptide, *VF* ventricular fibrillation, *HR* hazard ratio, *95% CI* 95% confidence interval. Model 1; multivariable model adjusting for age, log_e_-creatinine, first recorded heart rhythm and duration of resuscitation. Model 2; multivariable model adjusting for age, log_e_-creatinine, first recorded heart rhythm, duration of resuscitation, witnessed cardiac arrest and bystander-initiated cardiopulmonary resuscitation

Copeptin levels were elevated in all patients [436 (216–825) pmol/L], and did not differ between the two outcomes, *p* = 0.38. In survival analysis, copeptin quartiles did not separate survivors and non-survivors after 30 days, log-rank test *p* = 0.67. There were no statistically significant associations between copeptin levels and all-cause mortality at 30-days follow-up, neither in univariate analysis, nor in multivariable models (Table 2).

**Fig. 2:**
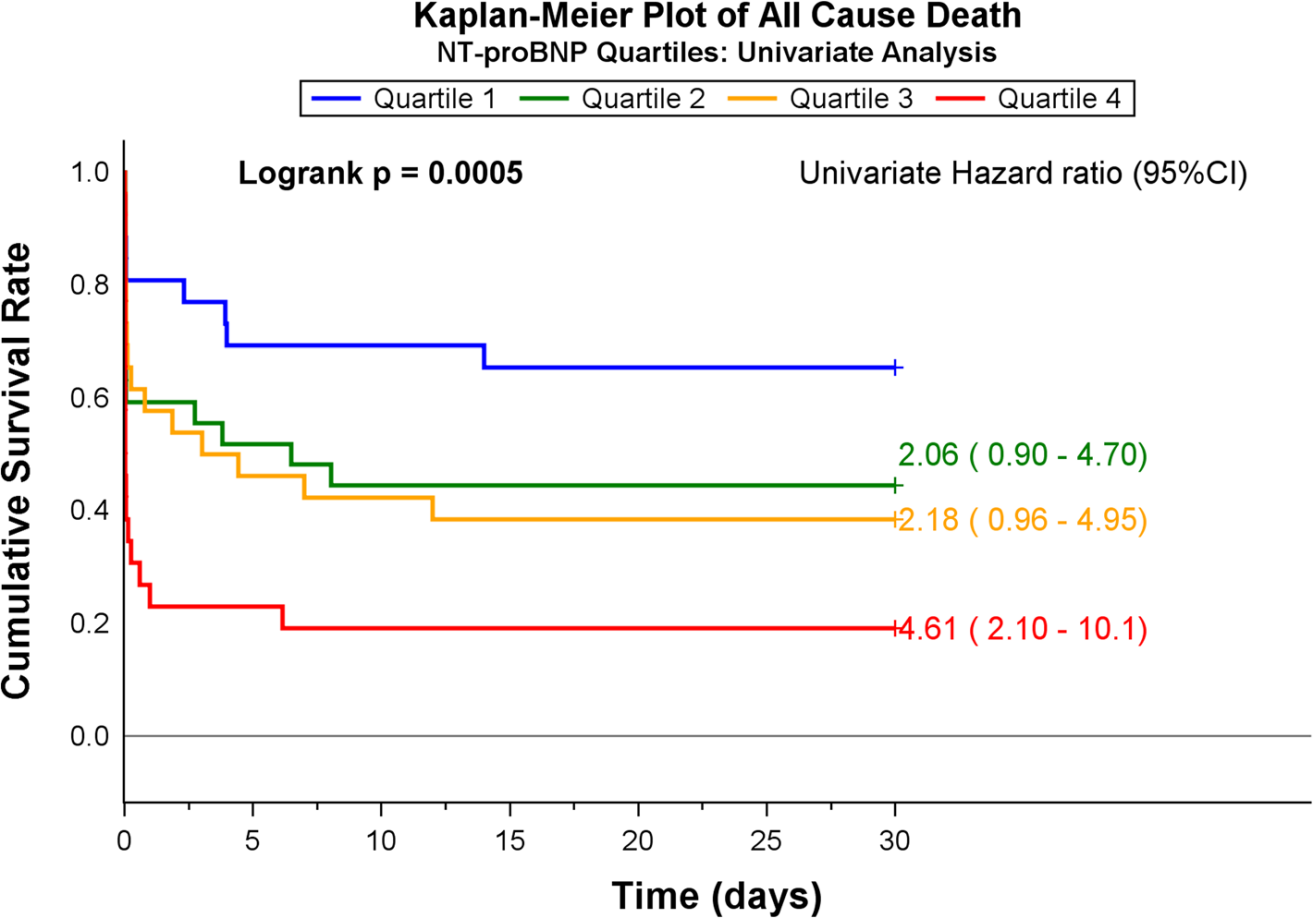
Survival curves up to 30-days in OHCA-patients stratified by NT-proBNP quartiles

**Fig. 3:**
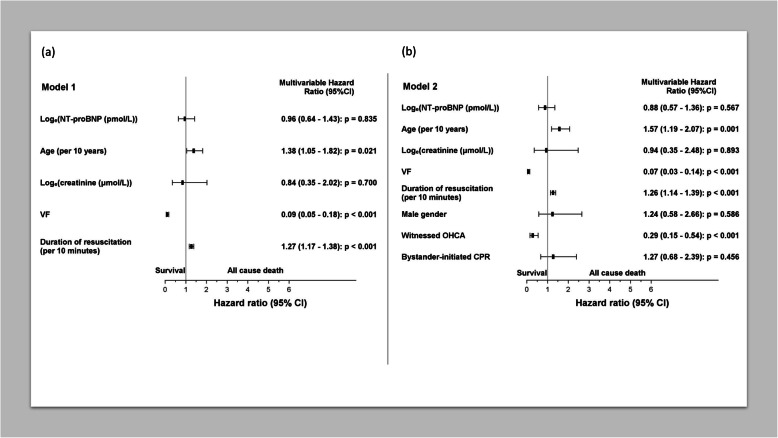
Adjusted hazard ratio and 95% confidence intervals for 30-day all-cause mortality according to selected risk factors from two different multivariable models. **a** Model 1, **b** Model 2. Hazard ratio (squares), 95% confidence interval (lines). Abbreviations: OHCA, out-of-hospital cardiac arrest; CPR, cardiopulmonary resuscitation; 95% CI, 95% confidence interval

NT-proBNP was the only biomarker demonstrating a significant difference between non-survivors and survivors [105 (35–495) pmol/L vs. 30 (12–98) pmol/L, *p* <  0.001]. Patients were grouped into quartiles according to NT-proBNP levels; Quartile 1 (Q1): < 24 pmol/L, Q2: 25–60 pmol/L, Q3: 62–218 pmol/L, and Q4: > 250 pmol/L. The median NT-proBNP level in Q4 was 720 (IQR; 371–2506). Q4-patients were significantly older with increased prevalence of heart failure and reduced kidney function, and there was a larger proportion with asystole as first recorded heart rhythm, Supplemental Table 1. Kaplan-Meier analysis revealed that the upper NT-proBNP quartile was significantly associated with all-cause mortality at 30-days (Fig. 2). Assessed as a continuous variable, this gave a univariate HR of 1.86, *p* <  0.001 for the total population, with similar findings in survivors at hospital admission [HR 1.86, *p* = 0.003] and in VF-patients [HR 1.67, *p* = 0.023]. Adding age, log_e_-creatinine value, VF as primary heart rhythm and duration of resuscitation in a multivariable Cox regression model (Model 1), resulted in loss of the significant association between NT-proBNP and outcome, both in the total population [HR 0.96, *p* = 0.84] (Fig. 3) and in the former two subgroups (Table 2). Similar results were obtained when adding gender, witnessed cardiac arrest and bystander-initiated CPR in multivariable model 2 (Table 2).

Among the clinical variables, increasing age was associated with death in both models (Model 2; *p* = 0.001). Witnessed cardiac arrest, VF as first recorded heart rhythm and duration of resuscitation were also significantly associated with outcome (Fig. 3).

NT-proBNP was significantly higher in patients who died on-scene (non-ROSC patients) compared to survivors at hospital admission [106.2 (46.5–706.2) pmol/L vs. 46.8 (17.2–164.4), *p* = 0.003], but there was no significant difference in NT-proBNP levels when comparing on-scene non-ROSC patients with those who died in hospital (*p* = 0.17). Hs-cTnT and copeptin did not differ significantly between on-scene non-ROSC patients and survivors at hospital admission.

At 30-day follow-up, all patients in the asystole-group had died, as compared to 43% in the VF-group. Baseline characteristics of patients with asystole versus VF as first recorded heart rhythm are presented in Table 3. NT-proBNP was the only biomarker that differed between these two groups, with a significantly higher median value of 250 (42–1305) pmol/L in the asystole group compared to 51 (18–111) pmol/L in the VF-group, *p* <  0.001. This difference is driven by VF-patients presenting with ischemic cardiac arrest (68.8% of the VF-population), *p* <  0.001, and was not evident when comparing non-ischemic cardiac patients with the asystole group, *p* = 0.74. Hs-cTnT and copeptin were elevated in both asystole- and VF patients, but there was no significant difference between the two groups. These findings are illustrated by applying log_e_-transformed values of the biomarkers as shown in Suppl. Fig. 1.

**Table 3:** Baseline characteristics and laboratory values of patients suffering out-of-hospital cardiac arrest, arranged according to first recorded heart rhythm

	**Asystole-patients (*n*** **= 37)**	**VF-patients (*n*** **= 77)**	***P*-value**
Age, y	75 (66–86)	63 (54–75)	< 0.001
Male sex	26 (70)	69 (90)	0.015
Death at 30 days	37 (100)	33 (43)	< 0.001
Died on scene	26 (70)	9 (12)	
Died in hospital	11 (30)	24 (31)	
**Cardiac arrest conditions**			
Witnessed cardiac arrest	26 (70)	61 (80)	0.24
Bystander-initiated CPR	26 (70)	67 (87)	0.040
Duration of resuscitation, min	28.5 (20–35)	15 (6–43)	0.082
**Previous history**			
Angina pectoris	9 (32)	8 (12)	0.037
Myocardial infarction	11 (32)	22 (30)	0.82
Previous PCI	4 (12)	8 (11)	1.00
Previous CABG	5 (15)	7 (10)	0.51
Heart failure	11 (33)	17 (23)	0.34
Hypertension	21 (64)	32 (46)	0.097
Diabetes mellitus	7 (21)	10 (14)	0.40
Hypercholesterolemia	12 (36)	32 (44)	0.53
Smoking			0.56
Current smoker	10 (39)	17 (29)	
Ex-smoker	10 (39)	30 (51)	
**Baseline blood samples**			
Creatinine (μmol/L)	101 (92–130)	102 (87–121)	0.42
Total cholesterol (mmol/L)	4.1 (3.5–5.0)	4.3 (3.7–5.4)	0.16
CRP (mg/L)	12.0 (2.0–50.0)	1.9 (1.1–4.1)	< 0.001
Glucose (mmol/L)	10.7 (3.7–15.7)	13.7 (9.6–17.2)	0.034
Copeptin (pmol/L)^a^	402 (244–939)	453 (210–765)	0.82
hs-cTnT (ng/L)^b^	67 (28–226)	73 (26–231)	0.77
NT-proBNP (pmol/L)^c^	250 (42–1305)	51 (18–111)	< 0.001

Data are presented as median (interquartile range) or numbers (%)

^a^ *n* = 110 (96%), ^b^ *n* = 111 (97%), ^c^ *n* = 112 (98%)

*Abbreviations*: *VF* ventricular fibrillation, *OHCA* out-of-hospital cardiac arrest, *CPR* cardiopulmonary resuscitation, *PCI* percutaneous coronary intervention, *CABG* coronary artery bypass graft, *CRP* C-reactive protein, *hs-cTnT* high-sensitivity cardiac troponin T

*NT-proBNP* N-terminal pro-B-type natriuretic peptide

## Discussion

We evaluated the prognostic utility of early-on levels of hs-cTnT, copeptin and NT-proBNP for all-cause mortality at 30-days follow-up in clinically characterized patients with OHCA of assumed cardiac origin, presenting with asystole or VF.

NT-proBNP was found to be significantly related to death in univariate analysis, whereas hs-cTnT and copeptin were not associated with outcome. Adjusting for demographic- and clinical variables, the prognostic value of NT-proBNP was attenuated and no longer statistically significant.

These findings are consistent with two previous studies [24, 25], reporting a similar association between admission levels of NT-proBNP and outcome in resuscitated OHCA patients. There are several possible mechanisms for increased NT-proBNP/BNP secretion during cardiac arrest and CPR, including hypoxemia [30], ischemia [31], ischemia-reperfusion induced inflammation [32], therapeutic interventions with administration of fluids [33] and vasopressors [34], and mechanical ventilation with supplementary oxygen administration [35]. Myhre et al. 2016 [24] demonstrated an increase in NT-proBNP levels from admission up to 96 h after hospitalization in OHCA-VT/VF patients. Longer time to ROSC and higher admission levels of hs-cTnT were found to be associated with high NT-proBNP concentrations after 24 h in multivariable analysis. Furthermore, 24-h NT-proBNP levels provided additional prognostic information for the prediction of 1-year mortality. These associations may reflect myocardial changes brought about by the cardiac arrest, whereas earlier on-site levels of NT-proBNP in our study most likely will reflect the pre-cardiac arrest condition, as patients in Q4 as compared to lower quartiles were more prone to heart failure, were older and presented with worse renal function. Accordingly, NT-proBNP levels did not differ between on-scene non-ROSC patients and those who died in hospital. The prognostic value of comorbidity has previously been claimed to be of less importance in OHCA patients [36, 37], and this may explain the lack of prognostic utility for early-on NT-proBNP in our study. However, early measurements of NT-proBNP may help to identify OHCA-patients with clinically silent heart insufficiency or coronary artery disease, in need of special medical attention.

Demographic- and clinical factors, such as age, witnessed cardiac arrest, bystander-initiated CPR, primary heart rhythm and time to ROSC, are previously found to be prognostically important in cardiac arrest [3, 38–42]. Furthermore, plasma concentrations of NT-proBNP are known to be associated with age and kidney function [13]. Multivariable analysis, adjusting for these established risk-variables, cancelled the significant univariate association between early-on levels of NT-proBNP and outcome. However, in accordance with previous observations [3, 39–42], we also noted that increasing age and longer duration of CPR was significantly associated with increased risk of death, while witnessed cardiac arrest and VF as primary heart rhythm was associated with a more favourable outcome (Fig. 3).

In contrast to previous reports [3, 39], bystander-initiated CPR was not found to be an independent outcome-predictor in our study, probably cancelled by a larger proportion of VF-patients and a short EMS response-time between 8 to 15 min in our recruitment area.

Furthermore, we found that NT-proBNP differentiated between ischemic VF patients and those presenting with asystole. This could be explained by a difference in baseline risk variables, as previously reported [21]. Patients in the asystole group were older and a higher proportion suffered from prior cardiac morbidity, including established coronary artery disease and heart failure. However, no difference in NT-proBNP was observed when comparing non-ischemic VF patients with those presenting with asystole. These groups had similar baseline characteristics and differed mainly by the presenting arrhythmia, which may relate to prognosis. Accordingly, all patients in the asystole group died as compared to 50% in the non-ischemic VF group.

The two other biomarkers, hs-cTnT and copeptin, were not related to outcome, neither in univariate, nor in multivariable analysis. As previously demonstrated [9], we found that essentially all resuscitated OHCA-patients had elevated levels of hs-cTnT. Gilje et al. [20] found that hs-cTnT peaked 24 h after admission following OHCA, but only hs-cTnT at 48 and 72 h, respectively, was independently associated with all-cause mortality. In our study, we analysed only one blood sample very close to the cardiac arrest, which may not reflect the peak values of hs-cTnT, and the prognostic utility may have been missed. This assumption is supported by the FINNRESUSCI substudy by Røsjø et.al [9], where admission levels of hs-cTnT failed to differ between hospital non-survivors and survivors and did not yield independent prognostic information at 1-year follow up in OHCA-VF/VT patients.

Copeptin levels were markedly increased in all patients in our study and did not differ between 30-days non-survivors and survivors. Admission levels of copeptin have previously been demonstrated to independently predict organ dysfunction and death in the ICU following OHCA [22]. As a predictor of long-term mortality, copeptin levels at day 3 were shown to perform better than copeptin measured at ICU-admission [23]. In our early-on sample, elevated copeptin may largely reflect the stress response during cardiac arrest, rather than outcome related hemodynamic instability following ROSC. The prognostic utility of copeptin claimed in the FINNRESUSCI population [22], is based on samples harvested up to 6 h after admission and did not include on-scene non-ROSC patients.

## Strengths

Blood was collected very early after OHCA and includes non-admitted patients without ROSC, a patient category usually missed out in previous studies. Also, the initial cardiac rhythm was recorded in all patients. Pre-hospital data were collected in accordance with the Utstein guidelines [26], and advanced cardiac life support was performed by the EMS paramedics according to current guidelines [27].

## Limitations

The small study population is one of the limitations. Further studies with larger patient populations should be performed to assess the prognostic value of these three biomarkers. Inclusion of patients was restricted to the largest ambulance centres in the area located closest to the hospital and to the medical support helicopter to ensure timely delivery of blood samples, limiting the potential recruitment area. Furthermore, patient recruitment could only be performed when there was enough EMS crew present at the OHCA-scene. Samples were obtained during resuscitation and not after death was declared. Unfortunately, there was a selection bias due to unbalanced blood sampling in the ROSC and non-ROSC group of patients. A few patients lacked detailed information regarding the OHCA. Our study is limited to 30-days observations of outcome.

## Conclusions

Early-on levels of hs-cTnT, copeptin and NT-proBNP did not provide prognostic information following OHCA. Early measurement of NT-proBNP seems to reflect the pre-cardiac arrest condition, and may be useful to identify OHCA-patients with heart insufficiency and coronary artery disease, also including subjects with clinically silent conditions.

## Supplementary information

**Additional file 1:** **Supplemental figure.**

**Additional file 2: Table S1.** Baseline characteristics and laboratory values of patients suffering out-of-hospital cardiac arrest, arranged according to Quartiles of NT-proBNP. Data are presented as median (interquartile range) or numbers (%). ^a^ *n* = 110 (96%), ^b^ *n* = 111 (97%), ^c^ *n* = 112 (98%). Abbreviations: OHCA, out-of-hospital cardiac arrest; CPR, cardiopulmonary resuscitation; PCI, percutaneous coronary intervention; CABG, coronary artery bypass graft; CRP, C- reactive protein; hs-cTnT, high-sensitivity cardiac troponin T; NT-proBNP, N-terminal pro-B-type natriuretic peptide.

## Data Availability

Local database. The datasets analysed during the current study are available from the corresponding author on reasonable request.

## Abbreviations

ACS: Acute coronary syndrome
AMI: Acute myocardial infarction
CI: Confidence interval
CPR: Cardiopulmonary resuscitation
cTn: Cardiac troponin
EDTA: Ethylenediamine tetra acetic acid
EMS: Emergency medical services
HR: Hazard ratio
Hs-cTnT: High-sensitivity cardiac troponin-T
ICU: Intensive care unit
IQR: Interquartile range
NT-proBNP: N-terminal pro-B-type natriuretic peptide
OHCA: Out-of-hospital cardiac arrest
ROSC: Return of spontaneous circulation
VF: Ventricular fibrillation
