# Supplementary figures and images for: N-terminal pro-B-type natriuretic peptide as a prognostic indicator for 30-day mortality following out-of-hospital cardiac arrest: a prospective observational study
Source: BMC Cardiovasc Disord. 2020 Aug 24;20:382. doi: 10.1186/s12872-020-01630-x (PMC7445901; doi:10.1186/s12872-020-01630-x)

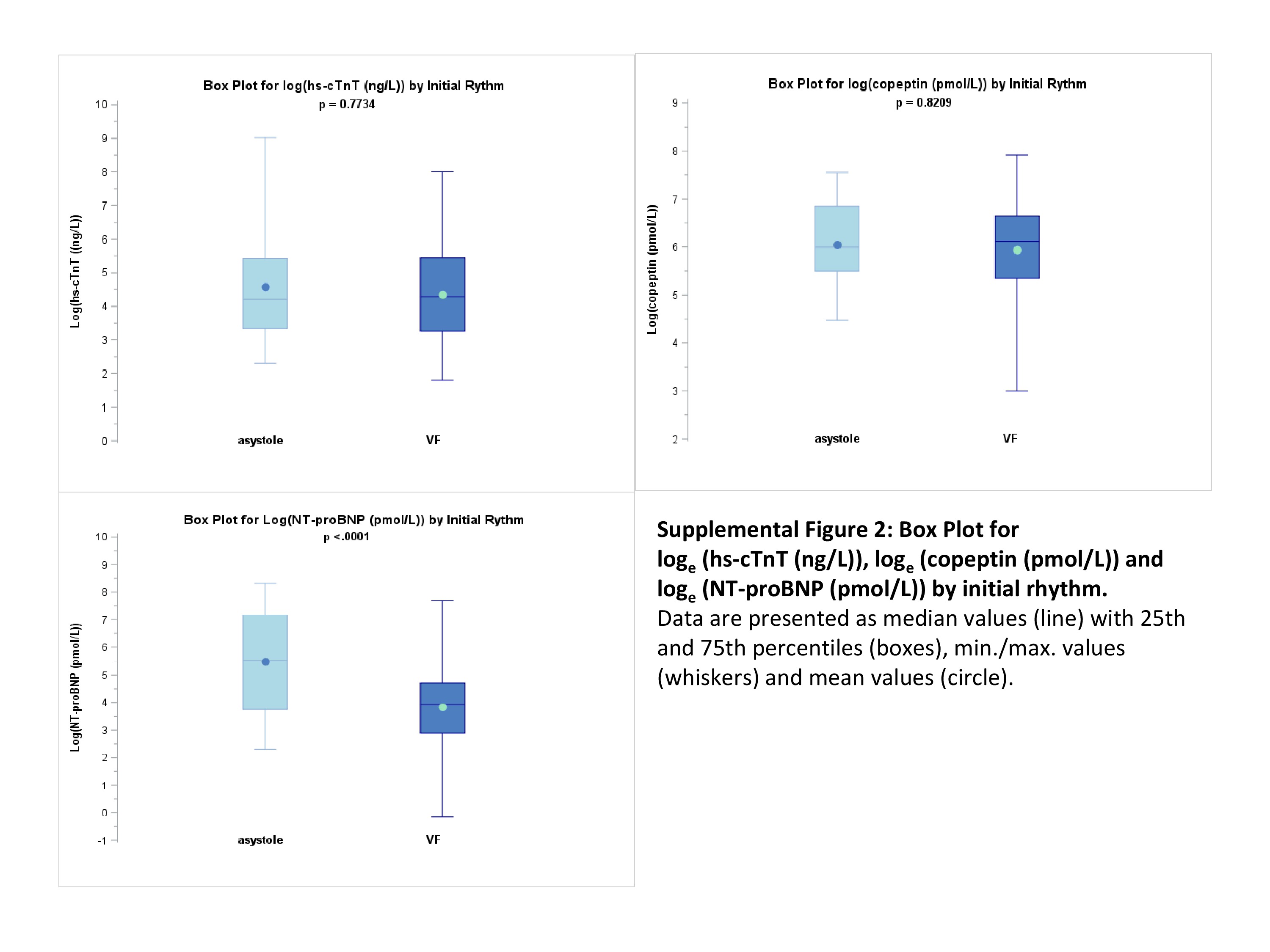

Supplement: Supplementary file 1 — Additional file 1: Supplemental figure. [file 12872_2020_1630_MOESM1_ESM.jpg]
