# Supplementary material for: N-terminal pro-B-type natriuretic peptide as a prognostic indicator for 30-day mortality following out-of-hospital cardiac arrest: a prospective observational study
Source: BMC Cardiovasc Disord. 2020 Aug 24;20:382. doi: 10.1186/s12872-020-01630-x (PMC7445901; doi:10.1186/s12872-020-01630-x)
Supplement: Supplementary file 2 — Additional file 2: Table S1. Baseline characteristics and laboratory values of patients suffering out-of-hospital cardiac arrest, arranged according to Quartiles of NT-proBNP. Data are presented as median (interquartile range) or numbers (%). a n = 110 (96%), b n = 111 (97%), c n = 112 (98%). Abbreviations: OHCA, out-of-hospital cardiac arrest; CPR, cardiopulmonary resuscitation; PCI, percutaneous coronary intervention; CABG, coronary artery bypass graft; CRP, C- reactive protein; hs-cTnT, high-sensitivity cardiac troponin T; NT-proBNP, N-terminal pro-B-type natriuretic peptide. [file 12872_2020_1630_MOESM2_ESM.docx]

**Supplemental Table 1: Baseline characteristics and laboratory values of patients suffering out-of-hospital cardiac arrest, arranged according to Quartiles of NT-proBNP.**

|  | **NT-proBNP Quartiles** | | | | |
| --- | --- | --- | --- | --- | --- |
|  | **Quartile 1**  **(N = 28)** | **Quartile 2**  **(N = 28)** | **Quartile 3**  **(N = 28)** | **Quartile 4**  **(N = 28)** | **P-value** |
| Age, y | 53 (47 - 62) | 68 (60 - 79) | 65 (57 - 78) | 78 (69 - 87) | < 0.001 |
| Male sex | 24 (86) | 23 (82) | 24 (86) | 22 (79) | 0.95 |
| Death at 30 days |  |  |  |  | 0.007 |
| Died on scene | 4 (14) | 9 (32) | 6 (21) | 16 (57) |  |
| Died in hospital | 7 (25) | 7 (25) | 12 (43) | 7 (25) |  |
| **Cardiac arrest conditions** |  |  |  |  |  |
| Initial rhythm |  |  |  |  | < 0.001 |
| Asystole | 4 (14) | 8 (29) | 5 (18) | 18 (64) |  |
| Ventricular fibrillation | 24 (86) | 20 (71) | 23 (82) | 10 (36) |  |
| Witnessed OHCA | 22 (79) | 24 (86) | 18 (64) | 21 (78) | 0.33 |
| Bystander-initiated CPR | 23 (82) | 23 (82) | 25 (89) | 20 (71) | 0.45 |
| Duration of resuscitation,  (min.) | 15.0 (6.0 - 44.0) | 15.0 (5.0 - 35.0) | 37.0 (18.0 - 64.0) | 25.0 (15.0 - 33.0) | 0.043 |
| **Previous history** |  |  |  |  |  |
| Angina pectoris | 4 (16) | 3 (13) | 3 (14) | 7 (32) | 0.39 |
| Myocardial infarction | 5 (19) | 7 (28) | 10 (36) | 11 (41) | 0.35 |
| Previous PCI | 4 (15) | 1 (4) | 4 (14) | 3 (11) | 0.58 |
| Previous CABG | 1 (4) | 2 (8) | 4 (14) | 5 (19) | 0.36 |
| Heart failure | 1 (4) | 3 (12) | 9 (33) | 15 (58) | < 0.001 |
| Hypertension | 11 (44) | 14 (56) | 11 (42) | 17 (68) | 0.23 |
| Diabetes mellitus | 2 (8) | 9 (35) | 1 (4) | 5 (19) | 0.016 |
| Hypercholesterolemia | 14 (54) | 11 (44) | 10 (37) | 9 (36) | 0.56 |
| Smoking |  |  |  |  | 0.26 |
| Current smoker | 12 (50) | 5 (29) | 4 (19) | 5 (23) |  |
| Ex-smoker | 7 (29) | 10 (59) | 11 (52) | 12 (55) |  |
| **Baseline blood samples** |  |  |  |  |  |
| Creatinine (µmol/L) | 98 (84 - 117) | 102 (84 - 115) | 98 (88 - 113) | 126 (96 - 198) | 0.013 |
| Total cholesterol (mmol/L) | 5.1 (3.8 - 6.9) | 4.4 (4.0 - 5.2) | 4.1 (3.4 - 4.9) | 3.9 (3.1 - 4.9) | 0.022 |
| CRP (mg/L) | 1.7 (1.1 - 4.3) | 1.8 (1.0 - 4.6) | 2.0 (1.1 - 6.1) | 14.5 (3.8 - 51.0) | < 0.001 |
| Glucose (mmol/L) | 11.8 (8.0 - 16.8) | 13.5 (11.3 - 16.9) | 14.2 (8.2 - 19.4) | 12.2 (5.8 - 14.4) | 0.47 |
| Copeptin (pmol/L)^a^ | 461 (295 - 925) | 389 (216 - 882) | 366 (138 - 793) | 425 (286 - 688) | 0.58 |
| hs-cTnT (ng/L)^b^ | 36 (15 - 118) | 42 (23-197) | 127 (45 - 314) | 93 (57 - 254) | 0.011 |
| NT-proBNP (pmol/L)^c^ | 11 (7 - 16) | 36 (28 -51) | 105 (84 - 168) | 720 (371 - 2506) | < 0.001 |

Data are presented as median (interquartile range) or numbers (%).

^a^ n = 110 (96%), ^b^ n = 111 (97%), ^c^ n = 112 (98%).

Abbreviations: OHCA, out-of-hospital cardiac arrest; CPR, cardiopulmonary resuscitation; PCI, percutaneous coronary intervention; CABG, coronary artery bypass graft; CRP, C- reactive protein; hs-cTnT, high-sensitivity cardiac troponin T; NT-proBNP, N-terminal pro-B-type natriuretic peptide.
